# Rise of extremity fractures and sport accidents in children at 8–12 years and increase of admittance via the resuscitation room over a decade

**DOI:** 10.1007/s00068-021-01785-y

**Published:** 2021-09-14

**Authors:** M. Voth, K. Sommer, C. Schindler, J. Frank, I. Marzi

**Affiliations:** Department of Trauma, Hand and Reconstructive Surgery, University Hospital, Goethe University Frankfurt, Theodor-Stern-Kai 7, 60590 Frankfurt am Main, Germany

**Keywords:** Pediatric, Trauma, Injury, Emergency room

## Abstract

**Introduction:**

In an emergency department, the majority of pediatric trauma patients present because of minor injuries. The aim of this study was to evaluate temporal changes in age-related injury pattern, trauma mechanism, and surgeries in pediatric patients.

**Methods:**

This retrospective study included patients < 18 years of age following trauma from 01/2009 to 12/2018 at a level I trauma center. They were divided into two groups: group A (A: 01/2009 to 12/2013) and group B (B: 01/2014 to 12/2018). Injury mechanism, injury pattern, and surgeries were analyzed. As major injuries fractures, dislocations, and organ injuries and as minor injuries contusions and superficial wounds were defined.

**Results:**

23,582 patients were included (58% male, median age 8.2 years).

There was a slight increase in patients comparing A (*n* = 11,557) and B (*n* = 12,025) with no difference concerning demographic characteristics.

Significant more patients (A: 1.9%; B: 2.4%) were admitted to resuscitation room, though the number of multiple injured patients was not significantly different.

In A (25.5%), major injuries occurred significantly less frequently than in B (27.0%), minor injuries occurred equally. Extremity fractures were significantly more frequent in B (21.5%) than in A (20.2%), peaking at 8–12 years.

Most trauma mechanisms of both groups were constant, with a rising of sport injuries at 8–12 years.

**Conclusion:**

Although number of patients increases only slightly over a decade, there was a clear increase in major injuries, particularly extremity fractures, peaking at 8–12 years. At this age also sport accidents significantly increased.

At least, admittance to resuscitation room rose but without an increase of multiple injured patients.

## Introduction

Since 2010, injuries are the second-most frequent reason for hospitalization in children of small age and even the most common reason for hospitalization in children of school age.[1]

In addition, severe injuries are the main cause of death among children (> 1 year) and adolescents [1]. More than 10% of death in children were caused by trauma (accident, suizid, violence, etc.) [2].

The five main causes of death by trauma (nearly 66%) among children aged from 1 to 14 years are drowning, falls from great heights, burns, traffic accidents, and violence [3].

Ellsäßer et al., 2019 showed that the number of fatal child injuries has decreased in the recent years. Traffic accident prevention has achieved demonstrable success with a continuous decrease in fatal and serious injuries from road traffic accidents among children and adolescents over the past 15 years [1]. There are many published studies on multiple trauma in children, but it is still rare and accounts for only 5% of all severely injured trauma patients [4, 5].

However, in the emergency unit of a hospital, additionally to serious injuries, a substantial number of lighter mono- or combination-injuries are treated. In a previous study we showed that 75% of all pediatric trauma patients who were admitted to the emergency department (ED) following trauma revealed minor injuries [6]. Only 0.45% of them suffered a multiple trauma. [6]

There are only a few studies concerning injury mechanisms and injury pattern of pediatric trauma patients [7–10]. Unfortunately, all these studies only concentrated on one parameter (fractures of long bones or the injury mechanism) and did not give an overview also about minor injuries and the injury mechanism.

The aim of this study was to show changes in characteristics of pediatric trauma in terms of demographic data, injury patterns and mechanisms and the need for surgery from 2009 to 2018 in a level I trauma center.

## Methods

### Study design

This study was performed retrospectively at the Hospital of the J. W. Goethe University, Department of Trauma, Hand and Reconstructive Surgery, Goethe University Frankfurt, Frankfurt am Main, Germany, with the approval of the institutional ethics committee (130/15, amendment from 07/2019) and in accordance with the Declaration of Helsinki and STROBE guidelines (Strengthening the Reporting of OBservational studies in Epidemiology) [11].

### Patients

All patients in the age of 0 to 17 years presenting to our emergency department (ED) at University Hospital, Goethe University Frankfurt, Frankfurt am Main, Germany, following trauma in the period from 01/2009 to 12/2018 were enrolled. Patients presenting to our ED with contact to a doctor got a recording protocol and so the patients could be identified retrospectively.

Exclusion parameters were an age ≥ 18 years, the absence of an acute trauma mechanism, presentation in our outpatient department and conditions after surgery and infections.

Trauma mechanisms were defined as following: “fall” (from standing height), fall from great height (≥ 1,5 m) (“fall great height”), traffic crashes (“traffic”), injuries caused by sport activities (“sports”), injuries caused on playground (“playground”), injuries following assaults (“violence”), injuries caused by contusions, distorsions or impacts by striking against or being struck unintentionally by objects or persons (“impact”) and injuries caused by pathologic causes (“pathologic”).

Amputations, fractures, dislocations, and visceral organ injuries as well as significant head injuries (skull fractures, intracerebral, epidural or subdural hematomas) were considered as “major injury”. “Minor injuries” were defined as a contusion, distorsion, burns or simple wounds.

Concussions and head contusions as well as skin/scalp lacerations and little wounds were considered as “minor head injuries”.

Multiply injured patients were defined by an Injury Severity Score (ISS) ≥ 16 pts [12].

A surgical intervention in the ED is defined for every treatment which was conducted in local anesthesia or short anesthetic (for example: suture of little wounds, suture of extensor tendons, reconstruction of nail beds, reposition of dislocations or fractures etc.). Surgeries in the OR (operation room) were for example: osteosynthesis of fractures, amputations, suture of nerves, and procedures that needed general anesthesia.

The cohort of pediatric trauma patients was divided by time period of admittance to our ED: group A (A) with an admittance between 01/2009 and 12/2013 and group B (B) with an admittance between 01/2014 and 12/2018. For further analysis group A and B were divided into four subgroups according to age: group 1 (0–3 years, “toddler”), group 2 (4–7 years, “pre-school”), group 3 (8–12 years, “school-age”), and group 4 (13–17 years, “teenager”).

### Data collection and analysis

The patient’s characteristics and injury mechanism, injury pattern, admission to the resuscitation room (RR, “shock room”)—for the hemodynamic stabilization and diagnosing of the patient—as well as the need for surgery were obtained from the patient´s electronic files using the medical protocols. A computerized spreadsheet (Microsoft Excel 2010; Microsoft Corporation, Redmond, WA) was created to abstract the variables. Chi-squared or Fisher’s exact test was used to compare categorical variables depending on sample size; Mann–Whitney *U* test was used to compare continuous variables (median and IQR [interquartile range]). A *p* value of < 0.05 was considered statistically significant. All analyses were performed using Bias 11.04 © (Epsilon Verlag GbR 1989–2016, Germany) or GraphPad Prism 3.02 © (GraphPad Software Inc. San Diego, CA).

## Results

### Demographic results and injury characteristics

The demographic results and injury characteristics in pediatric trauma patients stratified by time period of admission are presented in Table 1.

**Table 1:** Demographic and injury characteristics in pediatric trauma patients stratified by the period of admission

	All patients (*n* = 23,582)	Group A (*n* = 11,557)	Group B (*n* = 12,025)	*p* value
Age, years (Median, IQR)	8.2 (3.8–12.4)	8.1 (3.8–12.5)	8.2 (3.9–12.4)	0.37
Sex (male, *n*, %)	13,778 (58.4%)	6805 (58.9%)	6973 (58.0%)	0.48
Admission to RR (*n*, %)	511 (2.2%)	217 (1.9%)	294 (2.4%)	0.004
ISS ≥ 16 (*n*, %)	98 (0.4%)	44 (0.4%)	54 (0.5%)	0.48
Major injuries (*n*, %)	6184 (26.2%)	2942 (25.5%)	3242 (27.0%)	0.04
Minor injuries (*n*, %)	17,398 (73.8%)	8615 (74.5%)	8783 (73.0%)	0.31
Surgery in the OR (*n*, %)	1549 (6.6%)	721 (6.2%)	828 (6.9%)	0.06
Surgical intervention in the ED (*n*, %)	991 (4.2%)	379 (3.3%)	612 (5.1%)	< 0.001

Age is reported as median (interquartile range, IQR). All other values are reported as numbers and percentages

Group A: 2009–2013; Group B: 2014–2018

*ED* emergency department; *IQR* interquartile range; *OR* operating room; *RR* resuscitation room

Overall, 23,582 patients were included. Over time, there was a slight increase in the number of patients comparing groups A (*n* = 11,557) and B (*n* = 12,025) though this finding is not significant, *p* = 1.0.

The gender distribution was evident in both groups with nearly 60% male to 40% female patients (ratio 3:2), *p* = 0.48. The median age in both groups was 8.2 years (A: IQR (interquartile range): 3.8–12.5 years; B: IQR: 3.9–12.4 years; *p* = 0.37).

No significant difference in the number of patients with multiple injuries (A: *n* = 44; 0.4% and B: *n* = 54; 0.5%; *p* = 0.42) and in the Injury Severity Score (ISS) was found (A: ISS 22 pts. [IQR = 16.8–29 pts.] vs. B: ISS 25 pts. [IQR = 20–32 pts.]; *p* = 0.23). Nevertheless, in group B (*n* = 294; 2.4%), significantly more patients were admitted to the resuscitation room (RR) compared to A (*n* = 217; 1.9%), *p* < 0.05.

Overall, 6184 patients (26.2%) suffered a major and 17,398 patients (73.8%) a minor injury.

In group A (*n* = 2942, 25.5%), major injuries occurred significantly less frequently than in group B (*n* = 3242; 27.0%), *p* < 0.05. There was no significant difference in the frequency of minor injuries between the two groups (A: *n* = 8615 [74.5%] vs. B: *n* = 8783 [73%]), *p* = 0.3.

A total of 2540 patients (10.8%) required a surgical treatment. Of these, 1549 patients (6.6%) were operated in the operation room (OR) and 991 (4.2%) patients underwent a surgical intervention in the ED.

Comparing the two groups, in group B there were a slightly increase of surgeries in the OR (B: *n* = 828, 6.9% > A: *n* = 721, 6.2%; *p* = 0.06) and a significant increase of surgical interventions in the ED (B: *n* = 612, 5.1% > A: *n* = 379, 3.3%; *p* < 0.05).

### Injury pattern

#### Major injuries

Table 2 delineates the injury characteristics of pediatric trauma patients with a major injury.

**Table. 2:** Distribution of the major injuries stratified by the two patient groups

Injury	Group A (*n* = 11,557)	Group B (*n* = 12,025)	*p* value
Extremity fracture (*n*, %)	2139 (%)	2426 (%)	0.04
Chassaignac (*n*, %)	348 (%)	353 (%)	0.74
Dislocations (*n*, %)	124 (%)	128 (%)	0.95
Amputation (*n*, %)	38 (%)	31 (%)	0.31
Nerve/tendon/ligament (*n*, %)	103 (%)	178 (%)	< 0.0001
Head (*n*, %)	133 (%)	105 (%)	0.03
Thorax (*n*, %)	30 (%)	49 (%)	0.05
Abdomen (*n*, %)	19 (%)	23 (%)	0.63
Spine (*n*, %)	38 (%)	38 (%)	0.86

Values are reported as numbers and percentages

Major injuries were significantly more common in B with 3242 patients (27.0%) compared to A with 2942 patients (25.5%), *p* < 0.05. The predominating injuries in both groups were extremity fractures.

Comparing the different time groups, fractures were significantly more frequent in B (*n* = 2585, 21.5%) than in A (*n* = 2329, 20.2%), *p* < 0.05. In particular, the occurrence of fractures of the upper and lower extremities was significantly higher in B (*n* = 2426, 20.2%) compared to A (*n* = 2139, 18.5%), *p* < 0.05. The ratio of fractures of the upper extremity (A: 73.9% vs. B: 73.4%) to fractures of the lower extremity (A: 26.4% vs. B: 27.1) is comparable.

Subgroup analyses showed a significant higher occurrence of major injuries at the age of 8–12 years, particularly to fractures of the extremities (group 3 of A: *n* = 713 [6.2%] and group 3 of B: *n* = 879 [7.3%]), *p* < 0.05.

Furthermore, there was a significantly increase of injuries of nerves/tendons/ligaments in B (*n* = 178, 1.5%) compared to A (*n* = 103, 0.9%), *p* < 0.05. Subgroup analyses showed a significant increase in these injuries in group 2 (A: *n* = 10; B: *n* = 30), in group 3 (A: *n* = 34; B: *n* = 57), and in group 4 (A: *n* = 49; B: *n* = 80), *p* < 0.05. In particular, injuries of ligaments increased in group 2 (A: *n* = 2; B: *n* = 26), in group 3 (A: *n* = 21; B: *n *= 45), and in group 4 (A: *n* = 24; B: *n* = 47), *p* < 0.05.

For toddlers (age 0–3 years), no difference comparing group A (*n* = 10) and group B (*n* = 11) was observed for injuries of nerves/tendons and ligaments.

Additionally, major injuries of the head significantly decreased from A (*n* = 133, 1.2%) to B (*n* = 105, 0.9%), *p* < 0.05.

There was no significant difference between the both groups with regard to the occurrence of dislocation, organ injury (except for the head) or amputation.

#### Localization of extremity fractures

The majority of fractures occurred in the upper extremity (A: *n* = 1581, 73.9% and B: *n* = 1780, 73.4%), *p* = 0.9. Fractures of the lower extremity accounted for less than 30% in both groups (A: *n* = 564, 26.4% and B: *n* = 657, 27.1%), *p* = 0.7.

**Fig. 1:**
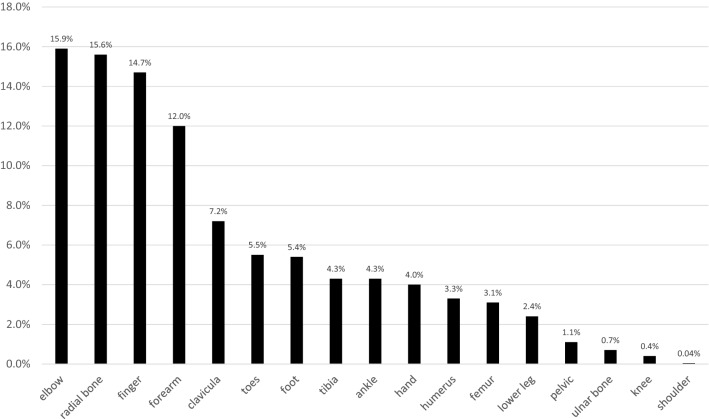
Fracture site in all pediatric trauma patients. Values are reported as percentages from all patients with fractures

Figure 1 outlines the localizations of the extremity fractures of all patients.

**Fig. 2:**
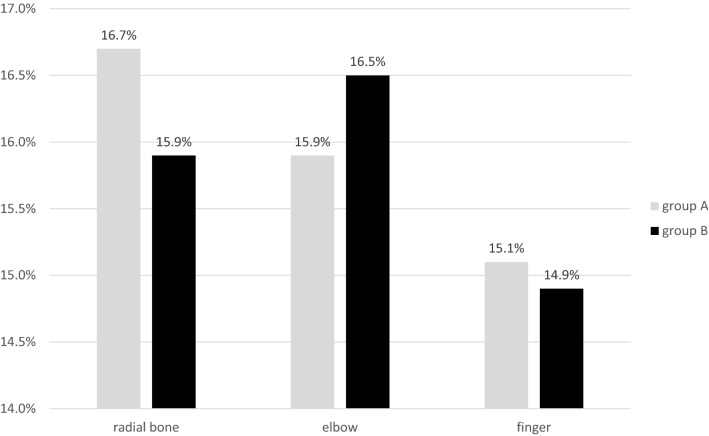
Three most localizations of the extremity fractures in groups A and B. Values are reported as percentages from all patients with extremity fractures

Most fractures were seen at the elbow joint (*n* = 740, 15.9%), followed by fractures of the radius (*n* = 726, 15.6%) and of the fingers (*n* = 684, 14.7%), Fig. 2.

In group A, the most presented fractures were radius fractures (16.7%), followed by fractures of the elbow (15.9%) and of the fingers (15.1%). In group B, the most frequent fractures were fractures of the elbow (16.5%), followed by radius fractures (15.9%) and finger fractures (14.9%).

In subgroup analyses of both groups, the elbow fracture was the most frequently found fracture in both main groups in children aged 4–7 years (group 2), followed by the group aged 8–12 years (group 3). With increasing age, the predominant fracture site in both main groups shifted to fractures of the forearm/radius (group 3) and to fractures of the finger (group 4).

#### Minor injuries

Overall, 17,398 patients (73.8%) suffered a minor injury. There was a slight increase in the number of minor injuries over time, but without significant difference (A: *n* = 8615, 74.5% and B: *n* = 8783, 73%), *p* = 0.3.

**Fig. 3:**
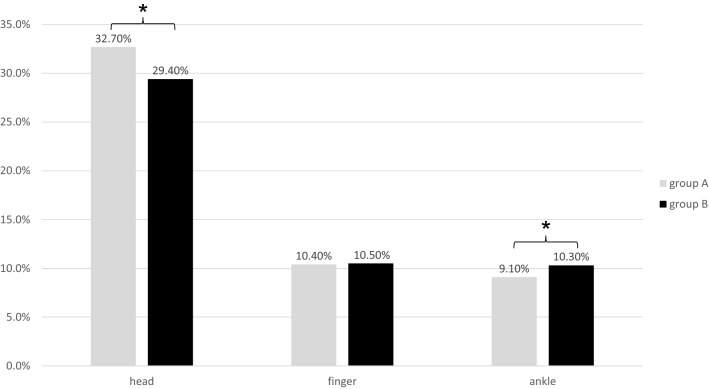
Three most frequently noticed localizations of minor injuries in group A and B. Values are reported as percentages from all patients with minor injuries. * *p* < 0.05: group A vs. group B

The most prevalent minor injuries of both groups were head injuries, injuries of the fingers and of the ankle (Fig. 3). There was a significant decrease of minor head injuries from A (*n* = 2820, 32.7%) to B (*n* = 2584, 29.4%) and a significant increase of ankle injuries from A (*n* = 787, 9.1%) to B (*n* = 901, 10.3%), *p* < 0.05. The occurrence of injuries of the fingers (A: *n* = 900 [10.4%], B: *n* = 918 [10.5%]) was not significantly different.

Subgroup analyses showed the highest incidence of minor head injuries in the age of 0–3 years (group 1) and 4–7 years (group 2) in both main groups [data not shown]. Furthermore, the highest incidence of injuries of the finger is in the age of 8–12 years (group 3) and of injuries of the ankle in the age of 13–17 years (group 4) in both main groups [data not shown].

### Injury mechanism

**Fig. 4:**
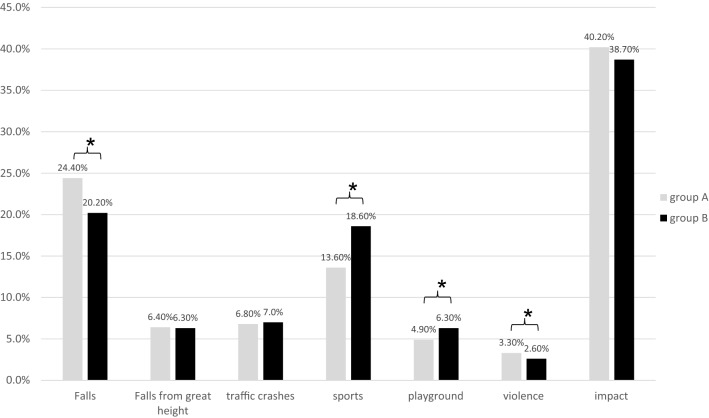
Trauma mechanism of all patients in groups A and B. Values are reported as percentages from all patients of groups A and B, respectively. * *p* < 0.05: group A vs. group B

Figure 4 shows the injury mechanism of all patients in groups A and B.

The most common trauma mechanism in both groups for all injuries was impact (A: 40.2%; B: 38.7%), followed by falls from standing height (A: 24.4%; B: 20.2%) and sports injuries (A: 13.6%; B: 18.6%).

The number of patients with falls (from standing height) or violence was significantly lower in B (20.2% and 2.6%, respectively) compared to A (24.4% and 3.3%, respectively). Otherwise, the number of patients suffering from sports accidents or injuries caused on playgrounds was significantly higher in B (18.6% and 6.3%, respectively) compared to A (13.6% and 4.9%, respectively). There was no significant difference between A and B concerning impact, fall from great height and traffic crashes. 1.4% of all fractures were pathological and similarly distributed between both patient groups.

The analyzes for major or minor injuries concerning trauma mechanism were the same like above mentioned and followed the same changes over time (data not shown).

In subgroup analysis, the incidence of injuries caused by falls (from standing height) and falls from great height was highest in group 1 (0–3 years) and 2 (4–7 years) in both main cohorts (A and B). In groups 3 (8–12 years) and 4 (13–17 years), injuries from violence, traffic accidents and sports had the highest incidence in both main groups. The highest incidence of injuries caused on playgrounds was found in groups 2 and 3 of both main groups. Injuries caused by impact had the highest incidence in groups 1 and 3 [data not shown].

Interestingly, in particular sports, injuries showed a high and significant increase at the age of 8–12 years (group 3) compared A (*n* = 596, 5.1%) to B (*n* = 984, 8.2%), *p* < 0.05.

### Surgery

Overall, 2540 patients (10.8%) required a surgical treatment. Of these, 1549 patients (6.6%) were operated in the OR and 991 (4.2%) patients underwent a surgical intervention in the ED.

**Fig. 5:**
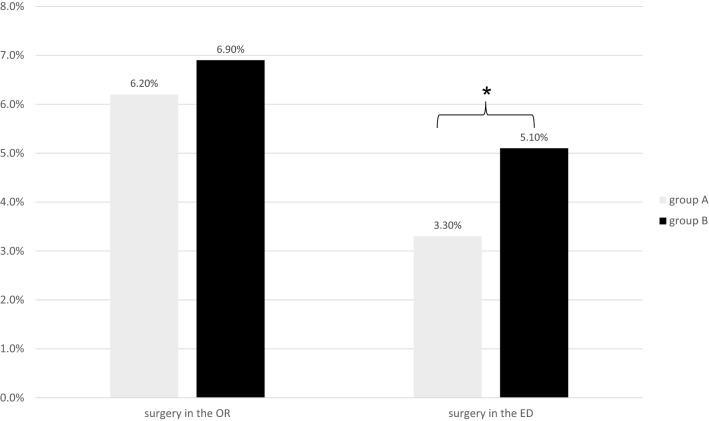
Surgical treatment of patients in groups A and B. Values are reported as percentages from all patients of groups A and B, respectively. ED: emergency department; OR: operation room. *p < 0.05: group A vs. group B

Comparing both groups, the number of operations in the OR was slightly higher in B (*n* = 828, 6.9%) compared to A (*n* = 721, 6.2%) without statistical significance (Fig. 5), *p* = 0.06.

Of all patients in group A undergoing surgery in the OR, 649 patients (90.0%) had a major injury and 72 patients (10.0%) had a minor injury. This is comparable to the 764 patients (92.3%) with a major injury and the 64 patients (7.7%) with minor injury in group B.

The most frequently found localizations of major injuries (mainly fractures and amputations) that were surgically treated in the OR were: elbow (A: 19.0%, B: 18.6%), forearm (A: 16.9%, B: 24.5%) and fingers (A: 15.1%, B: 11.9%). Thus, the surgical treatment in the OR of major injuries of the forearm was significantly increased in comparison of A to B (A: 16.9%, B: 24.5%), *p* < 0.05.

On the other hand, there also was a significant increase of surgical interventions in the ED compared group A (*n* = 379, 3.3%) to B (*n* = 612, 5.1%), *p* < 0.05. These surgical interventions in the ED were caused by major injuries (A: *n* = 45, 1.5% and B: *n* = 85, 2.6%)—like repositions of fractures or dislocations, reconstruction of nail beds, and suture of extensor tendons—as well as by minor injuries (A: *n* = 334, 3.9% and B: *n* = 527, 6.0%).

## Discussion

In the present retrospective analysis, 23,582 children were included over a study period of 10 years. To examine the changes over time in the epidemiology of pediatric trauma, these were divided into two time periods of 5 years each. Until now, only one other study has been published focusing on changes in injury patterns in pediatric patients over time. However, that study is focused on the pediatric upper extremity fracture incidence and does not look at all injuries in pediatric patients [13].

The number of patients enrolled in this study is similar to previously published studies of level I trauma centers in Germany [8]. The gender distribution was also comparable with other studies conducted so far in Germany, with a ratio of 3:2 (male:female ratio) [8, 10].

In the present study, a slight increase in the number of patients of about 4% was recorded over 10 years. This is comparable to another study that showed an annual increase in patient admittance in the emergency of 4–8% [14].

### Increased admittance to resuscitation room without increased number of multiple injured patients

Multiple trauma in children accounts for only 5% of all severely traumatized patients [5]. In the present study, 2.2% of all children were admitted to the resuscitation room (RR). This is markedly higher than recently reported by another trauma center in Germany (0.4%) [8]. In the present study, the number of patients admitted to the RR increased significantly over time. However, the number of multiple injured patients (ISS ≥ 16) did not differ over time.

This supports published data by Marzi et al. from 2019 which showed that the number of patients admitted to the RR increased over time, but the number of multiply traumatized patients is still the same [15]. So this shows an increasing “over-triage” of pediatric patients over the last years due to the changes of the prehospital algorithms. But it remains a matter of critical discussion whether this is not better than missing a relevant and life-threatening injury.

### Increased occurrence of major injuries

Nearly 75% of the patients included suffered a minor injury, which is comparable to the literature [8, 16, 17]. In the present study, the number of patients with minor injuries did not increase significantly. But there was a trend to an increase of major injuries over time. The predominating injuries were extremities fractures, mainly of the upper extremity, followed by Chassaignac lesions, luxations and injuries to nerves, tendons or ligaments. These results are comparable to data from all over the world. [8, 10, 18–20]

The number of fractures increased, with a consistent ratio of fractures of the upper to the lower extremity as well as most frequent fracture site. This contrasts with a recently published study from *Körner *et al*., 2019,* which showed a decrease in the absolute number of upper extremity fractures from 2002 to 2017 [13].

The incidence of childhood fractures peaks fairly consistent across the literature, with a peak around the age of 14 years for boys and 11 years for girls; thereafter, a decline is observed [21–25]. In our study, the peak of childhood fractures was around the age of 8 to 12 years. Furthermore, there was a significant increase in the occurrence of fractures in this age over the last 10 years. Thus, it seems that pediatric patients living in an urban setting such as ours suffer fractures in younger years (8–12 years) compared to those patients in countries in the cited studies. Furthermore, the increase of fractures in our study could be explain by an growing interest among children of extreme sports, such as skateboarding, snowboarding, and mountain biking, which are associated with an increased risk of fractures [26].

In the present study, there was a significant increase in injuries of nerves/tendons/ligaments over time in almost all age groups, except the age of 0–3 years. In particular, there was an increase of injuries of ligaments. Reasons for this could be, on the one hand, the well-known anatomical particularities in childhood such as higher elasticity of ligaments in early childhood and a decrease of this with increasing age, and, on the other hand, the increasing participation in road traffic and sport activities with increasing age.

### Decrease of major and minor head injuries and increase of minor injuries of the ankle

Concerning minor injuries, the most frequently found minor injury in this study was the head injury followed by injuries of the finger and ankle. Our previous study showed, that injuries to the head are the most common minor injury in all patient subgroups with a decrease observed from the age of 8 with increasing age because of the disproportionately large head in infants and toddlers combined with the still weak muscles and the lack of coordination.[6]

In contrast, minor injuries of the finger, hand or ankle increased with age, explained by increasing sport activities such as ballsports [8, 16].

In this study (major and minor), head injuries significantly decreased over the period of 10 years. A reason for this could be the prevention of falls in the age of infant and toddler (for minor head injuries) and the better accident prevention in the last years, e.g., in traffic accidents by wearing a helmet (for major head injuries) [1].

Minor ankle injuries increased in this study over time caused by the increased participation in sport activities with increasing age.

### Sport accidents rose over time, especially in the age of 8–12 years

In our study, the number of patients with sport accidents or injuries caused on playground was significantly increased. Interestingly, sport injuries showed a sharp and significant increase over time at the age of 8–12 years.

As previously described, this could be the reason for the increase in fractures and ligament injuries as well as minor injuries to the ankle at this age.

Concerning the other trauma mechanism, the most common injury mechanism was “impact”, followed by falls from standing height and sport accidents, which is comparable to *Albert *et al*., 2014*.[7]

Over time, there was no difference in the number of injuries caused by impact, fall from great height and traffic crashes in our study. Ellsäßer et al., 2019 showed, that there is a decrease of deadly injuries in children by a successful traffic accident prevention with a continuous reduction in fatal and serious injuries from traffic accidents but without varying the number of injuries caused by traffic crashes [2]. Reasons for the better outcome of children could be the better accident prevention, e.g., by wearing a helmet, the above-mentioned “over-triage” of the pediatric patients and the improved in-hospital treatment [1].

In addition, this study showed a significant reduction in injuries caused by falls or violence over a period of 10 years.

### Increase of number of operations and surgical interventions in the ED

10.8% of our patients required surgical treatment, with almost 7% needing surgery in the OR. This is much higher than previously reported by *Ruffing *et al*.* with only 3.1% patients requiring surgery but similar to our previous study [6, 8]. Since our level I trauma center covers hand and children´s orthopedic and general surgery, we are a primary referral center for pediatric patients with hand injuries and for multiply injured pediatric patients in particular.

This study found a slight increase in the number of operations over a 10-year period with a significant increase in the surgical treatment of major forearm injuries. With advancing age the bony spontaneous correction capability decreases, leading to a higher rate of operative interventions in older children. As previously shown, the number of fractures of the forearm increased with age, especially in the age of 8–12 years. This could possibly caused by a higher incidence of sports accidents in this age over time like previously shown. This could, therefore, be the reason for the increase in surgically treatment of major injuries of the forearm.

Furthermore, this study showed a significant increase of surgical interventions in the ED of major injuries—especially repositions of fractures or dislocations, reconstruction of nail beds and suture of extensor tendons—as well as of minor injuries over time. These findings are comparable to *Schlegel et al.* who also showed an increase of operative interventions in general [27].

### Limitations

The present study has several limitations. First of all, it is a retrospective study. Furthermore, it is not a real epidemiological study because only patients treated in our ED were included and other pediatric trauma patients, treated in non-emergency health care facilities or in our outpatient clinic, are not included. Otherwise, pediatric trauma patients were included who were sent in our ED from established doctors` practices.

## Conclusion

Over 5-year-periods of time, the number of pediatric patients increases only slightly, however, there was a clear increase in major injuries, particularly extremity fractures with a peak in the age of 8–12 years. At this age also sport accidents significantly increased over time, while major and minor injuries of the head decreased.

Although the most common trauma mechanisms are still the same, there is a trend to more sports accidents and injuries caused on playground and a decrease in injuries by falls from standing height or from violence. Whether this is due to social changes or reduced training of the children remains open. Number of operations slightly increased over time and surgical interventions in the emergency department rose significantly. The rise of an admittance of pediatric patients via the resuscitation room but without an increase of multiple injured pediatric patients indicates rather a logistic prehospital change.

## Abbreviations

A: Group A
B: Group B
ED: Emergency department
Fall: Fall from standing height
Fall great height: Fall from great height
Impact: Injuries caused by contusions, distorsions or impacts by striking against or being struck unintentionally by objects or persons
IQR: Interquartile range
ISS: Injury Severity Score
OR: Operation room
Pathologic: Injuries caused by pathologic causes
Playground: Injuries caused on playground
RR: Resuscitation room
Sports: Injuries caused by sport activities
Traffic: Traffic crashes
Violence: Injuries following assaults

## Declarations

### Availability of data and material

The submitted work is original and had not been published elsewhere in any form or language (partially or in full), unless the new work concerns an expansion of previous work.

## References

[1] Ellsäßer DMG. Unfälle, Gewalt, Selbstverletzung bei Kindern und Jugendlichen 2014. Ergebnisse der amtlichen Statistik zum Verletzungsgeschehen 2012. Fachbericht. Statistisches Bundesamt W, editor. wwwdestatisde. 2014.

[2] Ellsäßer G, Statistisches Bundesamt W. Unfälle, Gewalt, Selbstverletzung - Tabellenband - Ergebnisse der amtlichen Statistik zum Verletzungsgeschehen 2018. wwwdestatisde. 2019.

[3] Injuries in the European Union, summary on injury statistics 2012–14. EuroSafe A, editor. wwweurosafeeucom. 2016.

[4] Meier R, Krettek C, Grimme K, Regel G, Remmers D, Harwood P, et al. The multiply injured child. Clin Orthop Relat Res. 2005: 127–131. 10.1097/01.blo.0000156005.01503.0a 15738812

[5] Auner B, Marzi I (2014). Pediatric multiple trauma. Chirurg.

[6] Voth M, Lustenberger T, Auner B, Frank J, Marzi I (2017). What injuries should we expect in the emergency room?. Injury.

[7] Albert M, McCaig LF (2014). Injury-related emergency department visits by children and adolescents: United States, 2009–2010. Acad Emerg Med.

[8] Ruffing T, Danko S, Danko T, Henzler T, Winkler H (2015). Verletzungen bei Kindern und Jugendlichen im Bereitschaftsdienst. Dtsch Arztebl.

[9] Snyder CW, Muensterer OJ, Sacco F, Safford SD (2014). Paediatric trauma on the Last Frontier: an 11-year review of injury mechanisms, high-risk injury patterns and outcomes in Alaskan children. Int J Circumpolar Health.

[10] Kraus R, Schneidmüller D, Röder C (2005). Häufigkeit von Frakturen der langen Röhrenknochen im Wachstumsalter. Dtsch Arztebl.

[11] Elm von E, Altman DG, Egger M, Pocock SJ, Gøtzsche PC, Vandenbroucke JP. The Strengthening the Reporting of Observational Studies in Epidemiology (STROBE) Statement: Guidelines for reporting observational studies. Elsevier; 2014;12:1495–1499. Available from: http://www.journal-surgery.net/article/S1743-9191(14)00212-X/abstract

[12] Baker SP, O'Neill B, Haddon W, Long WB (1974). The injury severity score: a method for describing patients with multiple injuries and evaluating emergency care. J Trauma.

[13] Körner D, Gonser CE, Bahrs C, Hemmann P (2020). Change in paediatric upper extremity fracture incidences in German hospitals from 2002 to 2017: an epidemiological study. Arch Orthop Trauma Surg.

[14] Riessen R, Gries A, Seekamp A, Dodt C, Kumle B. Positionspapier für eine Reform der medizinischen Notfallversorgung in deutschen Notaufnahmen. Springer. 2015 10.1007/s00063-015-0050-y 26024948

[15] Marzi I, Lustenberger T, Störmann P, Mörs K. Steigender Vorhalteaufwand für den Schockraum. Springer. 2019 10.1007/s00113-018-0484-9 29556688

[16] Jonasch E, Bertel E. Verletzungen bei Kindern bis zum 14. Lebensjahr. 1981;. 7239942

[17] Kahl H, Dortschy R, Ellsäßer G (2007). Verletzungen bei Kindern und Jugendlichen (1–17 Jahre) und Umsetzung von persönlichen Schutzmaßnahmen. Bundesgesundheitsblatt - Gesundheitsforschung - Gesundheitsschutz.

[18] Randsborg P-H, Gulbrandsen P, Benth JS, Sivertsen EA, Hammer O-L, Fuglesang HFS (2013). Fractures in children: epidemiology and activity-specific fracture rates. J Bone Joint Surg Am.

[19] Park MS, Chung CY, Choi IH, Kim TW, Sung KH, Lee SY (2013). Incidence patterns of pediatric and adolescent orthopaedic fractures according to age groups and seasons in South Korea: a population-based study. Clin Orthop Surg.

[20] Joeris A, Lutz N, Wicki B, Slongo T, Audigé L (2014). An epidemiological evaluation of pediatric long bone fractures - a retrospective cohort study of 2716 patients from two Swiss tertiary pediatric hospitals. BMC Pediatr.

[21] Kopjar B, Wickizer TM (1998). Fractures among children: incidence and impact on daily activities. Inj Prev.

[22] Cooper C, Dennison EM, Leufkens HGM, Bishop N, van Staa TP (2004). Epidemiology of childhood fractures in Britain: a study using the general practice research database. J Bone Miner Res.

[23] Naranje SM, Erali RA, Warner WC, Sawyer JR, Kelly DM (2015). Epidemiology of pediatric fractures presenting to emergency departments in the United States. J Pediatr Orthop.

[24] Hedström EM, Svensson O, Bergström U, Michno P (2010). Epidemiology of fractures in children and adolescents. Acta Orthop.

[25] Clark EM (2014). The epidemiology of fractures in otherwise healthy children. Curr Osteoporos Rep.

[26] Mathison DJ, Agrawal D. An update on the epidemiology of pediatric fractures. journalslwwcom. 2010. 10.1097/PEC.0b013e3181eb838d 20693861

[27] Schlegel C, Greeno A, Chen H, Raees MA, Collins KF. Evolution of a level I pediatric trauma center: changes in injury mechanisms and improved outcomes. Elsevier. 2018. 10.1016/j.surg.2017.10.070 29373171

